# Hepatocyte-specific deletion of cellular repressor of E1A-stimulated genes 1 exacerbates alcohol-induced liver injury by activating stress kinases

**DOI:** 10.7150/ijbs.67852

**Published:** 2022-01-31

**Authors:** Miaomiao Wu, Fan Yin, Xiaoli Wei, Ruixue Ren, Chongqing Chen, Menghua Liu, Ruyu Wang, Liu Yang, Ruiqian Xie, Shanyue Jiang, Ziming Wang, Rui Liu, Wentao Xu, Xuefu Wang, Jing Li, Hua Wang

**Affiliations:** 1 School of Pharmacy, Anhui Medical University, Hefei, 230032, China.; 2 Department of Oncology, the First Affiliated Hospital of Anhui Medical University, Hefei, 230022, China.; 3 Inflammation and Immune Mediated Diseases Laboratory of Anhui Province, Anhui Medical University, Hefei, 230032, China.; 4 School of Life Sciences, Anhui Medical University, Hefei, Anhui 230032, China.

**Keywords:** CREG1, ALD, Apoptosis, Steatosis, Inflammation

## Abstract

Alcohol-associated liver disease (ALD) encompasses a wide range of pathologies from simple steatosis to cirrhosis and hepatocellular carcinoma and is a global health problem. Currently, there are no effective pharmacological treatments for ALD. We have previously demonstrated that aging exacerbates the pathogenesis of ALD, but the underlying mechanisms are still poorly understood. Cellular repressor of E1A-stimulated genes 1 protein (CREG1) is a recently identified small glycoprotein that has been implicated in aging process by promoting cellular senescence and activating stress kinases. Thus, the current study aimed to explore the role of aging associated CREG1 in ALD pathogenesis and CREG1 as a potential therapeutic target. Hepatic and serum CREG1 protein levels were elevated in ALD patients. Elevation of hepatic CREG1 protein and mRNA was also observed in a mouse model of Gao-binge alcohol feeding. Genetic deletion of the *Creg1* gene in hepatocytes (*Creg1^∆hep^*) markedly exacerbated ethanol-induced liver injury, apoptosis, steatosis and inflammation. Compared to wild-type mice, *Creg1^∆hep^* mice had increased phosphorylation of hepatic stress kinases such as apoptosis signal-regulating kinase 1 (ASK1), c-Jun N-terminal kinase (JNK) and p38 but not TGF-β-activated kinase 1 (TAK1) or extracellular signal-regulated kinase (ERK) after alcohol feeding. *In vitro*, ethanol treatment elevated the phosphorylation of ASK1, JNK, and p38 in mouse hepatocyte AML-12 cells. This elevation was further enhanced by CREG1 knockdown but alleviated by CREG1 overexpression. Last, treatment with an ASK1 inhibitor abolished ethanol-induced liver injury and upregulated hepatic lipogenesis, proinflammatory genes and stress kinases in *Creg1^∆hep^* mice. Taken together, our data suggest that CREG1 protects against alcoholic liver injury and inflammation by inhibiting the ASK1-JNK/p38 stress kinase pathway and that CREG1 is a potential therapeutic target for ALD.

## Introduction

Alcohol-associated liver disease (ALD) is a major public health problem globally. In the earliest stages of ALD, hepatic steatosis appears to be reversible; however, chronic and excessive alcohol consumption may result in steatohepatitis, fibrosis, and, in some cases, cirrhosis and even hepatocellular carcinoma 1. Alcohol exposure triggers hepatocyte apoptosis and necrosis in hepatocytes as well as hepatic inflammation. The second impact of ethanol consumption is the disturbance of lipid metabolism, which results in excessive triglyceride (TG) accumulation in the liver, aggravating liver injury 2. Although disease progression is well described and understanding of its pathogenesis has been gained over the years, the exact pathogenic mechanisms remain obscure, and there are no FDA-approved therapies to prevent or cure the disease 3. Therefore, to develop rationally targeted therapies for prevention or treatment, it is necessary to gain a better understanding of the mechanisms that lead to ALD initiation and progression.

Cellular repressor of E1A-stimulated genes 1 (CREG1), a small glycoprotein, was initially identified as a transcription repressor that represses E1A-induced transcription activation, and subsequent studies have demonstrated that that CREG1 regulates senescence, autophagy, and mitogen-activated protein kinase (MAPK) signalling 4, 5. Aging is associated with progressive changes in hepatic structure and function, and also increases the risk of various types of liver diseases, including ALD, non-alcoholic liver disease, liver fibrosis and liver cancer 6. Our previous studies have shown that aged mice have a high susceptibility to ethanol-induced liver damage, mainly manifested by excessive inflammation and oxidative stress 7, 8. In addition, it has been documented that activation of stress kinases plays critical role in the progression of ALD 9. CREG1 has been implicated in the aging process and promotes cellular senescence by cooperating with CDKN2A/p16^ink4a^ 10. Furthermore, CREG1 is elevated in the serum and liver of aged mice, age-related obesity and renal dysfunction are ameliorated in adipocyte P2-CREG1 transgenic mice 11. Recent studies have shown that CREG1 promotes lysosomal biogenesis function and improves the skeletal muscle response to exercise endurance by regulating mitophagy 12, 13. In cardiovascular disease studies, the function of CREG1 primarily relies on its ability to inhibit inflammation, activate autophagy, and inhibit vascular smooth muscle cell death by modulating MAPK signalling 14-16. Additionally, in liver disease studies, CREG1 was reported to inhibit hepatic steatosis and metabolic disorders in high-fat-diet (HFD)-fed mice through direct interaction with apoptosis signal-regulating kinase 1 (ASK1) and inactivation of ASK1-c-Jun N-terminal kinase (JNK1) signalling; CREG1 was also found to protect against cell death and inflammation during hepatic ischemia-reperfusion (I/R) injury in a TGF-β-activated kinase 1 (TAK1)-dependent manner 17, 18. Aging and activation of stress kinases have been shown to play an important role in promoting ALD development 9. Given the multiple biological functions of CREG1, we hypothesized that CREG1 may be involved in regulating the pathogenesis of ALD. To test this hypothesis, we generated hepatocyte-specific *Creg1* knockout mice and utilized Gao-binge alcohol feeding models. Our results suggest that CREG1 protects against ALD by inhibiting pathways including the ASK1/JNK/p38 stress kinases.

## Materials and method

### Animal experiments

All animal experiments were approved by the Animal Ethics Committee of Anhui Medical University (AMU). Mice with hepatocyte-specific *Creg1* knockout were generated by crossing *Creg1* floxed mice (GemPharmatech Co., Ltd.) with *Alb*-Cre mice with both strains on the C57BL/6J background. All animals were housed in ventilated cages at the SPF facility of AMU in a temperature-controlled environment with a 12-hour light/dark cycle and had free access to a normal chow diet (70% carbohydrate, 20% protein, and 10% fat) and water.

For the ALD mouse models, we tested 2 ethanol feeding protocols modified from a previous report by Bin Gao's laboratory: i) the traditional Lieber-DeCarli diet feeding model (a long-term, chronic model; age-matched 8-week-old male mice were fed with either a 5% (vol/vol) alcohol-containing liquid diet or an isocaloric control diet for 5 weeks, hereafter referred to as “the tradition model”) 19, 20; and ii) the short-term Gao-binge ethanol feeding model (age-matched 8-week-old male mice were fed with either an isocaloric control or ethanol Lieber-DeCarli diet containing 5% (vol/vol) ethanol for 10 days. On day eleven, ethanol- and pair-fed mice were dosed by oral gavage with a single bolus of ethanol (5 g/kg body weight) and isocaloric maltose dextrin solution, respectively (This NIAAA model was also referred to as “the Gao-binge model”)^21^-^23^. The ASK1 inhibitor GS-4997 (3 mg/Kg GS-4997) was injected intraperitoneally daily into mice on day 4 of alcohol feeding for 7 consecutive days. At the end of the experiments, liver tissues and serum were collected for further analysis.

### Cell culture and treatment

The mouse hepatocyte AML-12 cell line was purchased from the Cell Bank of the Chinese Academy of Sciences. AML-12 cells were cultured in DMEM/F12(PM150310, Procell) supplemented with 10% foetal bovine serum and 1% antibiotics and incubated at 37 °C in a humidified atmosphere containing 5% CO2. Ethanol was added directly to the medium of the cells after they had grown to 70% confluency. The culture dishes were sealed with laboratory film throughout the treatment period to minimize the evaporation of ethanol, while nontreated AML-12 cells were used as a control.

### Human liver and serum samples

Human liver tissues from patients with ALD and controls were collected at the time of surgery. Sera were collected from intoxicated patients and healthy individuals. The information of the intoxicated patients and healthy individual are listed in Supplementary Table S1. The study protocol and the use of biosamples for research were approved by the Ethics Committee of the First Hospital of AMU.

### *Creg1* gene silencing and overexpression

The siRNAs and overexpression plasmids were purchased from Gene Pharma Corporation (Shanghai, China). AML-12 cells were transfected with CREG-siRNA or pEX3-CREG1 and their control constructs using LipofectamineTM 2000 (Invitrogen, Carlsbad, CA, USA) according to the manufacturer's protocol. The following siRNA sequences were used: CREG1-siRNA: 5'- CUGGUUCUUUGCUAAAUUATT-3' (sense) and 5'-UUCUUGAAGACCCUGGUCCTT-3' (antisense). The AML-12 cell line was used for overexpression and silencing experiments in the presence and absence of ethanol at the defined concentration.

### Biochemical assays

Mouse peripheral blood was collected and centrifuged to obtain serum for analysis. We used a Mindray automated biochemical analyser (BS-350E) to test the serum alanine aminotransferase (ALT), aspartate aminotransferase (AST), TG and total cholesterol (TC) levels.

### Hepatic TG and TC analysis

TG and TC concentrations were determined by commercially available assay kits (Nanjing Jiancheng Bioengineering Institute). The procedures were performed according to the manufacturer's instructions, and the absorbance was measured at 510 nm using a microplate reader (Bio-Rad Laboratories, Hercules, USA).

### Histopathological analysis and immunohistochemistry (IHC)

Fresh liver tissue was fixed in 10% formalin solution. Fixed liver tissues were embedded in paraffin, cut into 5 μm thick slices and stained with haematoxylin and eosin (HE) following the standard protocol for analysis. Liver tissues were perfused with freezing medium and then frozen at -80 °C. Subsequently, they were sliced for Oil Red O staining following the standard protocol. IHC staining for CREG1 (SC-100695, Santa Cruz), myeloperoxidase (MPO, ab208670, Abcam), and F4/80 (70076S, CST) was performed on paraffin-embedded tissue sections (5 μm), visualized using DAB substrate. Slices were scanned by an automatic digital slide scanner (Pannoramic MIDI, 3DHISTECH, Hungary) and analysed by CaseViewer software. We quantified and counted the positively stained areas and cells by using Image-Pro Plus (40× magnification) software in 3 fields randomly selected for each sample.

### Terminal deoxynucleotidyl transferase dUTP nick end labelling (TUNEL)

To detect individual apoptotic cells, staining for terminal deoxynucleotidyl transferase dUTP nick-end labelling (TUNEL) was carried out using a TUNEL Bright Green Apoptosis Detection Kit (Vazyme Biotech, Nanjing, China) according to the manufacturer's instructions. Samples were observed with an automatic digital slide scanner (Pannoramic MIDI, 3DHISTECH, Hungary) and analysed by CaseViewer software. The numbers of total and TUNEL-positive cells were counted and analysed using Image-Pro Plus (40× magnification) software.

### ELISA

Liver tissue was added to phosphate-buffered saline (PBS) containing protease inhibitors. The tissues were then homogenized for 60 sec. The homogenates were then centrifuged, and the supernatant was collected. Commercially available ELISA kits (Dakewei, Shanghai, China) were used to detect TNFα, IL-1β and IL-6 levels according to the instructions provided by the manufacturer.

### Protein isolation and Western blotting

Total protein extraction was performed with RIPA buffer to which protease inhibitors and phosphatase inhibitors had been added. We determined the concentration of total protein using the NanoDrop2000 system (Thermo Scientific, USA) following the manufacturer's instructions (Beyotime, Jiangsu, China). Protein samples were mixed with loading buffer and separated by 10% sodium dodecyl sulfate-polyacrylamide gel electrophoresis. Then, the proteins were transferred onto polyvinylidene difluoride membranes (PVDF; Millipore Corp, Billerica, MA, USA). PVDF membranes were blocked in TBST containing 5% skim milk for 2 h at room temperature. Primary antibodies were used for Western blotting. Afterwards, the blots were incubated for 1 h at room temperature with secondary antibodies of the respective species. Analyses were carried out using an ECL kit (ECL-plus, Thermo Scientific, USA) and ImageJ software to quantify signals. All of the information about the antibodies used is provided in Supplementary Table S2.

### RNA isolation and RT-qPCR analysis

Total RNA was extracted using TRIzol (Invitrogen). RNA was quantified by a Nanodrop2000 spectrophotometer (Thermo Scientific, USA). cDNA was synthesized using an RT-qPCR kit (Takara, QIAGEN, Japan) and detected using a Pikoreal 96 real-time PCR system (Thermo Scientific, USA). The relative mRNA expression levels were calculated using the 2 -ΔΔC method and were normalized against the levels of β-actin. The primer sequences used for each gene are listed in Supplementary Table S3.

### Statistical analysis

All data are expressed as the mean ± SEM. We analysed two samples using the t test, while multiple samples were analysed by using the Kruskal-Wallis test and one-way analysis of variance. GraphPad Prism 8.0 software was used to perform all relevant statistical analyses. In all cases, *p*<0.05 was considered statistically significant.

## Results

### Hepatic and Serum CREG1 levels are upregulated in patients and mice with ALD

**Figure 1:**
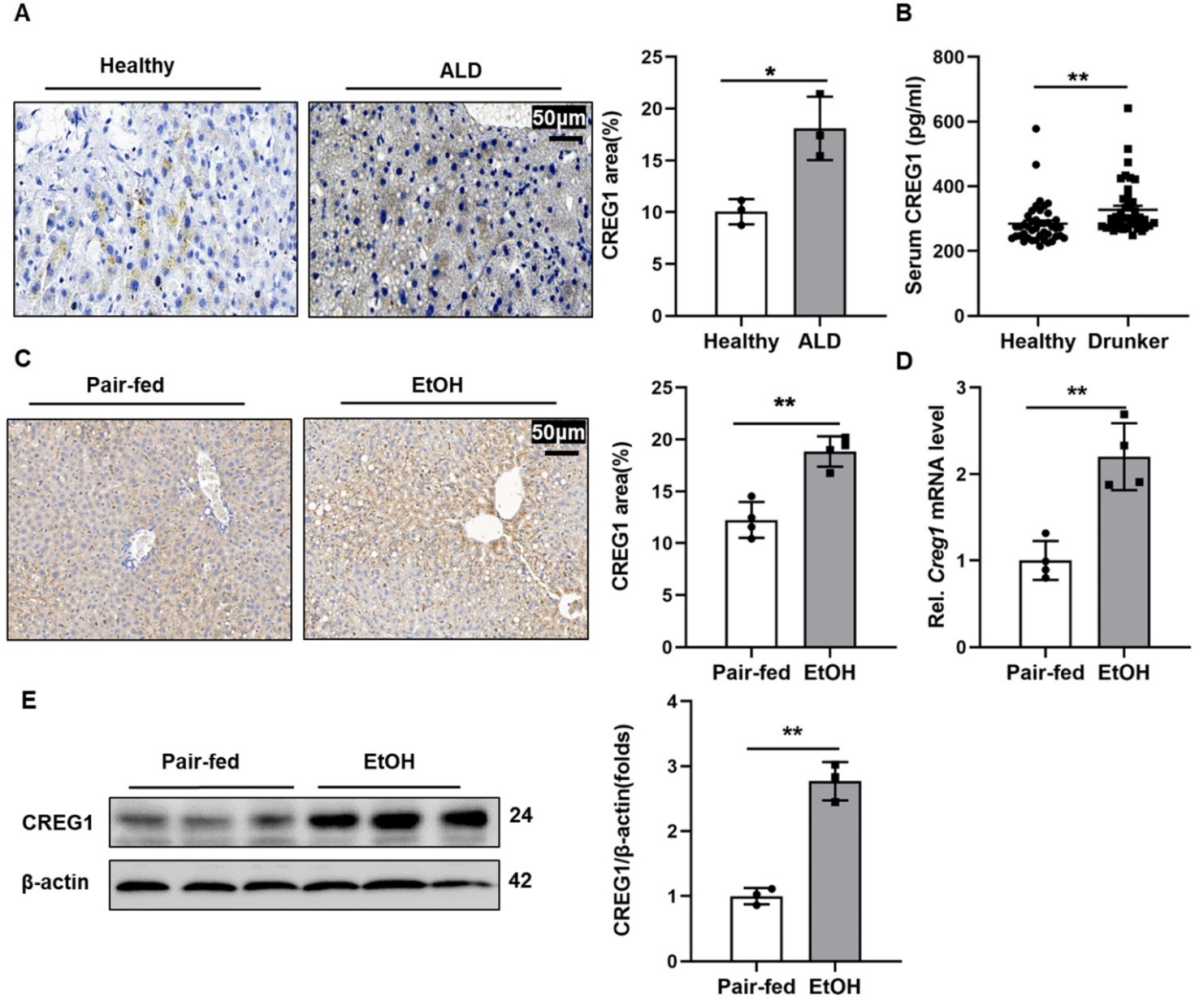
** Hepatic CREG1 expression levels are increased in ALD patients and ethanol-fed mice. (A)** Liver tissues were obtained from patients with ALD and healthy controls. Immunohistochemistry analysis of hepatic CREG1 expression (n=3 per group). Scale bars: 50 µm. **(B)** Serum was obtained from intoxicated individuals and healthy controls. ELISA analysis of CREG1 expression in serum (n=43 per group). **(C-E)** Mice were pair-fed or ethanol-fed for 10 days and administered a single binge of ethanol (Gao-binge model). **(C)** Immunohistochemistry assessment of CREG1 expression (n=4 per group). **(D)** mRNA levels of hepatic CREG1 (n=4 per group). **(E)** Western blotting to measure hepatic CREG1 expression (n=3 per group). β-Actin served as the loading control. All data are represented as the mean ± SD. *p < 0.05, **p < 0.01 by two-tailed Student's t test. CREG1, cellular repressor of E1A-stimulated genes 1.

To investigate the potential role of CREG1 in the progression of ALD, we first evaluated CREG1 expression levels in patients with ALD and in healthy controls. As shown in Figure 1A, IHC staining showed that CREG1 was significantly increased in the ALD patients. Additionally, the serum level of CREG1 was also elevated in drunk patients compared with healthy individuals (Figure 1B). Next, we established a short-term chronic plus one binge feeding model (Gao-binge model) (Supplementary Figure 1). Increased hepatic protein expression of CREG1 was also detected in ALD mice by IHC analyses (Figure 1C). Hepatic mRNA and protein levels of CREG1 were markedly upregulated in ALD mice compared to their counterparts (Figure 1D, E). CREG1 expression were also increased in AML12 cells after ethanol treatment (Supplementary Figure 2A, B). Overall, these results suggest that increased hepatic CREG1 expression is associated with ALD.

### Hepatocyte-specific *Creg1* deletion exacerbates ethanol-induced liver injury and apoptosis

**Figure 2:**
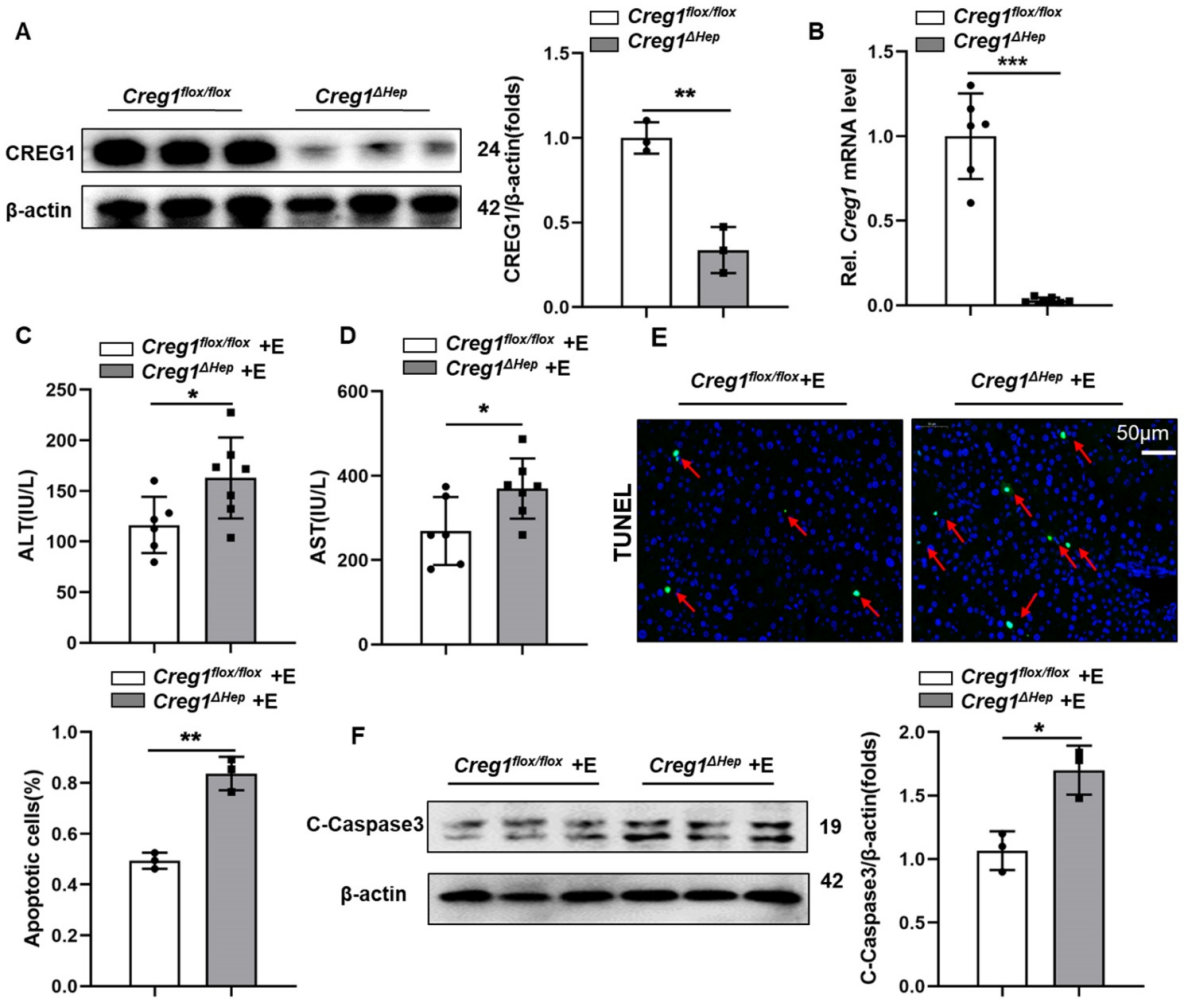
** Hepatic *Creg1* deletion promotes ethanol-induced liver injury and apoptosis. (A)** Protein and **(B)** mRNA levels of CREG1 in liver tissues from *Creg1^flox/flox^* and *Creg1^∆hep^* mice to confirm *Creg1* deletion in hepatocytes (n= 3-6 per group). (C-F) *Creg1^flox/flox^* and *Creg1^∆hep^* mice were ethanol-fed for 10 days and administered a single binge of ethanol (Gao-binge model). **(C)** The levels of ALT and **(D)** AST in the sera of *Creg1^flox/flox^* and *Creg1^∆hep^* mice after ethanol treatment (*n*=6-8 per group). **(E)** Representative images and quantification of TUNEL staining of liver sections (*n*=3 per group). Scale bars: 50 µm. **(F)** Levels of cell death-related protein c-Caspase3 expression (*n*=3 per group). β-Actin served as the loading control. All data are represented as the mean ± SD. *p < 0.05, **p < 0.01 by two-tailed Student's t test. ALT, alanine aminotransferase; AST, aspartate aminotransferase; HE, haematoxylin and eosin; TUNEL, terminal deoxynucleotidyl transferase dUTP nick end labelling.

Interestingly, CREG1 in Kupffer cells was not altered by alcohol consumption (Supplementary Figure 2C, D), so we focused more on the role of CREG1 in hepatocytes in ALD and generated hepatocyte-specific CREG1 knockout (Creg1∆hep) mice (Supplementary Figure 3). Western blotting and RT-qPCR analysis confirmed specific CREG1 deletion in hepatocytes (Figure 2A, B). We subjected aged-matched control *Creg1^flox/flox^* and *Creg1^∆hep^* mice to the Gao-binge model and assessed the ensuing alterations. Neither serum indices nor liver morphology differences were noted in *Creg1^∆hep^* mice versus *Creg1^floxl/flox^* mice in the pair-fed group (Supplementary Figure 4). In the ethanol-fed group, no differences were found between *Creg1^∆hep^* mice and *Creg1^floxl/flox^* mice in terms of body weight, liver weight, or liver weight-to-body weight ratio (Supplementary Figure 5A, B, C). To identify whether CREG1 affects alcohol metabolism, the levels of several enzymes associated with alcohol metabolism, such as cytochrome P450 2E1 (*Cyp2E1*), alcohol dehydrogenase 1 (*Adh1*), and alcohol dehydrogenase 2 (A*dh*2), were comparable between *Creg1^∆hep^* mice and *Creg1^floxl/flox^* mice after ethanol feeding (Supplementary Figure 5D, E). Compared to *Creg1^floxl/flox^* mice, ethanol-fed *Creg1^∆hep^* mice had higher serum ALT and AST levels (Figure 2C, D). Immunofluorescence staining showed that *Creg1^∆hep^* mice had more cell death (cells with TUNEL positivity) than their counterpart mice after ethanol feeding (Figure 2E). In addition, greater expression of pro-cell death proteins was observed in ethanol-fed* Creg1^∆hep^* mice than in *Creg1^floxl/flox^* mice (Figure 2F).

### Hepatocyte-specific *Creg1* deletion worsens ethanol-induced hepatic steatosis

**Figure 3:**
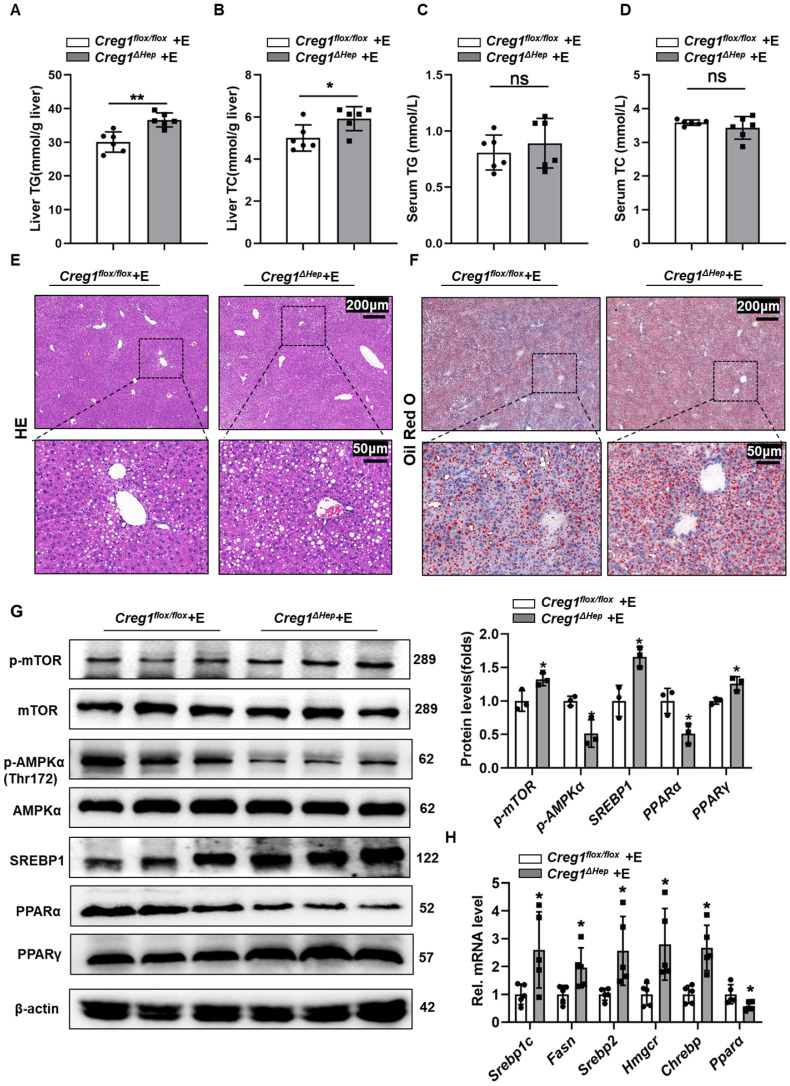
** Hepatic *Creg1* deletion aggravates ethanol-induced lipid metabolism disorders and hepatic steatosis in mice. (A-H)** *Creg1^flox/flox^* and *Creg1^∆hep^* mice were ethanol-fed for 10 days and administered a single binge of ethanol (Gao-binge model). **(A)** Hepatic triglyceride (TG) and **(B)** total cholesterol (TC) levels. **(C)** TG and **(D)** TC levels in serum (*n*=6 per group). **(E)** Representative haematoxylin and eosin (HE) staining of liver tissues. **(F)** Representative Oil Red O staining of liver tissues (*n*=3 per group). Scale bar = 200 µm; scale bar = 50 µm. **(G)** Levels of lipid synthesis- and lipid metabolism-related protein expression (*n*=3 per group). **(H)** Levels of lipid metabolism-related mRNA expression (*n*=5 per group). β-Actin served as the loading control. All data are represented as the mean ± SD. ns, not significant, *p < 0.05, **p < 0.01 by two-tailed Student's t test. TG, triglyceride; TC, total cholesterol. HE, haematoxylin-and eosin. *Srebp1-c,* sterol regulatory element binding protein, *Fasn,* fatty acid synthase, *Hmgcr,* 3-hydroxy-3-methyl-glutaryl-coenzyme a reductase, *Chrebp,* carbohydrate response element-binding protein, *Ppara,* peroxisome proliferator activated receptor alpha, *Pparg,* peroxisome proliferator activated receptor gamma, *Ampk*, AMP-activated protein kinase, *mTOR*, mammalian target of rapamycin.

In addition to apoptosis, lipid accumulation is also a key step in the development of ALD 3, 24. Therefore, we further determined the effect of CREG1 deficiency on hepatic steatosis. As shown in Figure 3A and Figure 3B, TG and TC levels in the liver were higher in ethanol-fed *Creg1^∆hep^* mice compared with those in *Creg1^flox/flox^* mice. However, there were no differences in serum TG and TC levels between these two groups (Figure 3C, D). HE and Oil staining demonstrated more lipid accumulation in the livers of ethanol-fed *Creg1^∆hep^* mice compared to these in ethanol-fed *Creg1^flox/flox^* mice (Figure 3E, F). To determine which signalling pathways were dysregulated to produce the alcoholic steatosis exacerbated by CREG1 deletion, we measured the expression of a number of proteins involved in lipid metabolism. The AMP-activated protein kinase (AMPK)/mammalian target of rapamycin (mTOR) pathway regulates many metabolic processes, such as glucose metabolism, lipid metabolism, and energy homeostasis 25. Western blotting analyses showed that p-mTOR, SREBP1, and peroxisome proliferator activated receptor gamma (PPARγ) were elevated while p-AMPK and PPARα were decreased in *Creg1^∆hep^* mice compared with *Creg1^flox/flox^* mice after ethanol feeding (Figure 3G). Furthermore, ethanol-fed *Creg1^∆hep^* mice showed increased expression of mRNAs associated with fatty acid generation (*Srebp1-c, Fasn, Srebp2, Hmgcr, and Chrebp*) and decreased mRNA levels of* Ppar-α* in liver tissue (Figure 3H). These results provide compelling evidence that CREG1 in hepatocytes protects against ethanol-induced lipid metabolism disorders and hepatic steatosis.

### Hepatic *Creg1* deficiency aggravates liver inflammation in ALD

**Figure 4:**
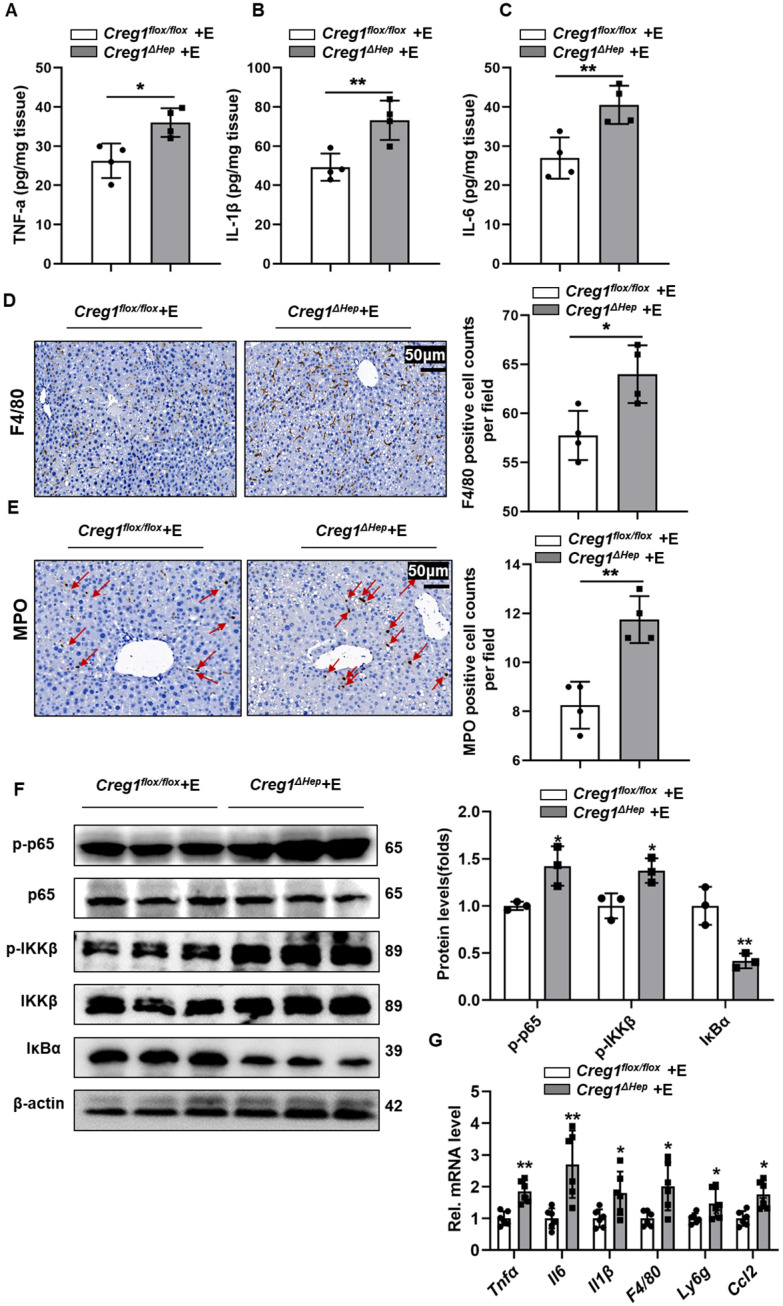
** Hepatic *Creg1* deletion aggravates inflammatory responses in ethanol-fed mice. (A-F)** *Creg1^flox/flox^* and *Creg1^∆hep^* mice were ethanol-fed for 10 days and administered a single binge of ethanol (Gao-binge model). The proinflammatory cytokines **(A)** TNFα, **(B)** IL-β and **(C)** IL-6 were tested by using ELISA kits (*n*=4 per group). **(D)** F4/80 immunohistochemistry staining as a marker of macrophages and **(E)** MPO immunohistochemistry staining as a marker of neutrophils (*n*=4 per group). Scale bar = 50 µm. **(F)** Western blotting analysis to evaluate the activation of NF-κB signalling (*n*=3 per group). **(G)** mRNA levels of proinflammatory (*Tnfα*, *Il6*, *Il1*, *F4/80*, *Ly6g*, and *Ccl2*) genes (*n*=6 per group). β-Actin served as the loading control. All data are represented as the mean ± SD. *p < 0.05, **p < 0.01 by two-tailed Student's t test.* Tnfa*, tumour necrosis factor alpha; *Il6*, interleukin 6; *Il1β*, interleukin 1 beta.

Inflammation is a hallmark of ALD 24. Proinflammatory cytokines in liver tissue, such as tumour necrosis factor alpha (TNF-α), interleukin 6 (IL-6) and interleukin 1 beta (IL-1β), were markedly increased in ethanol-fed *Creg1^∆hep^* mice compared to *Creg1^flox/flox^* mice (Figure 4A, B, C). Similarly, F4/80 and MPO staining showed more macrophage and neutrophil infiltration, respectively, in *Creg1^∆hep^* mice than in *Creg1^flox/flox^* mice following ethanol treatment (Figure 4D, E). The phosphorylation levels of IKKβ and p65 were significantly enhanced while that of IKBα was significantly decreased in ethanol-fed* Creg1^∆hep^* mice compared to ethanol-fed *Creg1^flox/flox^* mice (Figure 4F). In addition, hepatic mRNA levels of *Tnf-α*,* Il-6*, *Il-1β*, *F4/80*, and *Ly6g* and the secretion of C-C chemokine ligand 2 (*Ccl2*) were elevated in ethanol-fed* Creg1^∆hep^* mice compared to ethanol-fed *Creg1^flox/flox^* mice (Figure 4G). Overall, these data indicate that CREG1 in hepatocytes plays a key role against the inflammatory response in ALD.

### Hepatic CREG1 deficiency exacerbates hepatocyte apoptosis, hepatic steatosis and inflammation in long-term chronic alcohol consumption mice

**Figure 5:**
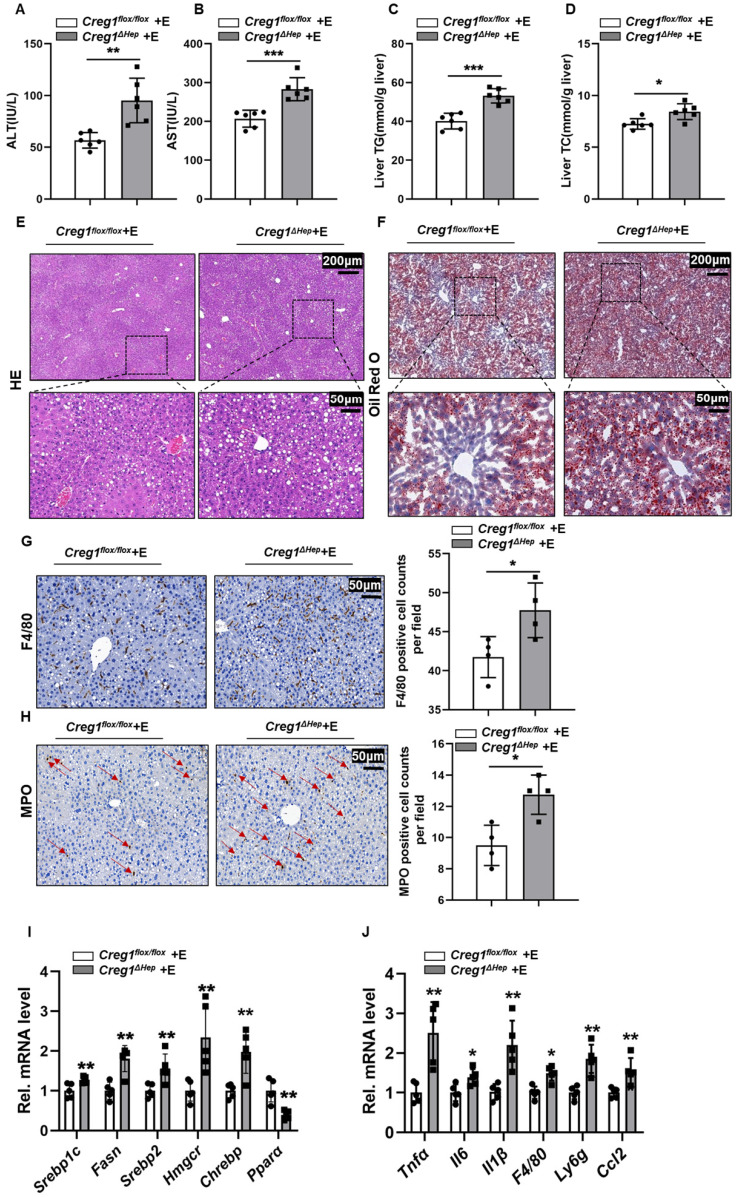
** Hepatic *Creg1* deficiency exacerbates apoptosis, steatosis and inflammation in mice in a traditional ALD model. (A-J)** *Creg1^flox/flox^* and *Creg1^∆hep^* mice were ethanol-fed for 5 weeks (traditional model). **(A)** The levels of ALT and (B) AST in the sera of *Creg1^flox/flox^* and *Creg1^∆hep^* mice after long-term chronic ethanol feeding (*n*=6 per group). **(C)** Hepatic triglyceride (TG) and **(D)** total cholesterol (TC) levels (*n*=6 per group). **(E)** Representative HE staining of liver tissues. **(F)** Representative Oil Red O staining of liver tissues. Scale bar = 200 µm; scale bar = 50 µm. **(G)** F4/80 staining and **(H)** MPO staining of liver sections (*n*=4 per group). Scale bar = 50 µm. **(I)** Levels of lipid metabolism-related and **(J)** proinflammatory mRNA expression (*n*=5 per group). All data are represented as the mean ± SD. *p < 0.05, **p < 0.01 by two-tailed Student's t test.

To further verify that CREG1 plays a protective role in ALD, another well-established, traditional ALD model was implemented. Under the traditional model, Wester blotting analysis showed that hepatic expression of CREG1 and CYP2E1 was elevated following ethanol treatment, whereas CYP2E1 expression did not differ in ethanol-fed *Creg1^flox/flox^* mice and *Creg1^flox/flox^* mice (Supplementary Figure 6). Compared with *Creg1^flox/flox^* mice, the serum ALT and AST levels were increased in *Creg1^∆hep^* mice after ethanol feeding (Figure 5A, B). Similarly, the levels of TG and TC were elevated in ethanol-fed *Creg1^∆hep^* mouse livers (Figure 5C, D). HE staining and Oil Red O staining showed that liver lipid accumulation was significantly increased in ethanol-fed *Creg1^∆hep^* mouse livers (Figure 5E, F). In addition, ethanol-fed *Creg1^∆hep^* mice also had more severe immune cell infiltration into the liver tissue (Figure 5G, H). The mRNA levels of molecules that promote fat accumulation and inflammation were increased in ethanol-fed *Creg1^∆hep^* mice compared with those in *Creg1^flox/flox^* mice (Figure 5I, J). These data suggest for the first time that CREG1 protects against alcohol-induced liver damage in two well-established animal models of ALD.

### CREG1 diminishes activation of ASK1-p38/JNK signalling

**Figure 6:**
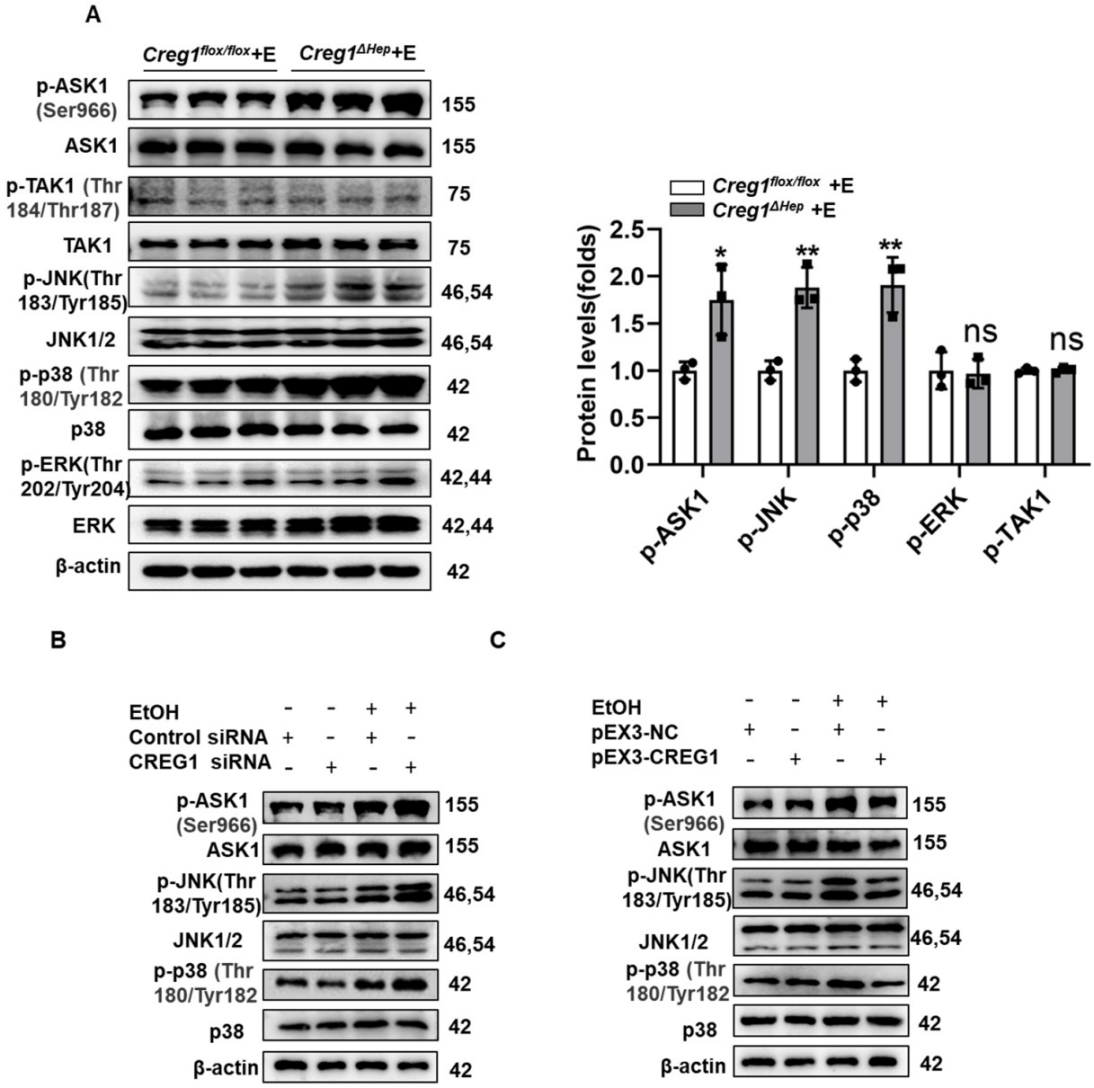
** CREG1 inhibits ethanol-induced activation of ASK1-JNK/p38 signalling. (A)** *Creg1^flox/flox^* and *Creg1^∆hep^* mice were ethanol-fed for 10 days and administered a single binge of ethanol (Gao-binge model). The protein levels of total and phosphorylated ASK1, TAK1, ERK, JNK, and p38 were assessed (n=3 per group). **(B)** AML-12 cells were transfected with or without CREG1 siRNA and subsequently treated with 100 mM ethanol and analysed by Western blotting for total and phosphorylated ASK1, JNK, and p38. **(C)** AML-12 cells were transfected with or without pEX3-CREG1 and subsequently treated with 100 mM ethanol and analysed by Western blotting for total and phosphorylated ASK1, JNK, and p38. β-Actin served as the loading control. All data are represented as the mean ± SD. *p < 0.05, **p < 0.01 by two-tailed Student's t test. ASK1, apoptosis signal-regulating kinase 1, JNK, c-Jun N-terminal kinase, TAK1, TGF-β-activated kinase 1, ERK, extracellular signal-regulated kinase.

Subsequently, considering the potent regulation of CREG1 in ALD, we investigated the underlying mechanisms by which CREG1 inhibits the progression of ALD. Activation of ASK1 and p38MAPK has been shown to play an important role in promoting ALD development 9. Additionally, numerous studies indicate that CREG1 mediates the stress kinase pathway, suggesting that it may regulate stress kinase signalling during ethanol-induced liver injury 14, 26, 27. As shown in Figure 6A, the phosphorylation of JNK and p38 was significantly increased in liver samples of *Creg1^∆hep^* mice compared with those of *Creg1^flox/flox^* mice after ethanol feeding. However, ERK phosphorylation was not affected by CREG1 modulation. To identify possible upstream molecules that contribute to the activation of JNK and p38, we examined the activation of TAK1 and ASK1. The results showed that phosphorylation of ASK1, but not that of TAK1, was significantly elevated in *Creg1^∆hep^* mice. To confirm the above results, cellular experiments were subsequently performed, and Western blotting and RT-qPCR were used to evaluate the efficiency of CREG1 silencing and overexpression (Supplementary Figure 7). CREG1 knockdown significantly increased the phosphorylation of ASK1, JNK and p38 (Figure 6B) in AML-12 cells treated with ethanol (100 mM), whereas CREG1 overexpression decreased the phosphorylation of ASK1, JNK and p38 (Figure 6C). Taken together, both the *in vivo* and *in vitro* data indicate that the ASK1-p38/JNK signalling pathway is regulated by CREG1 during ethanol-induced liver injury.

### CREG1 protects the liver from ethanol-induced liver injury by targeting ASK1

**Figure 7:**
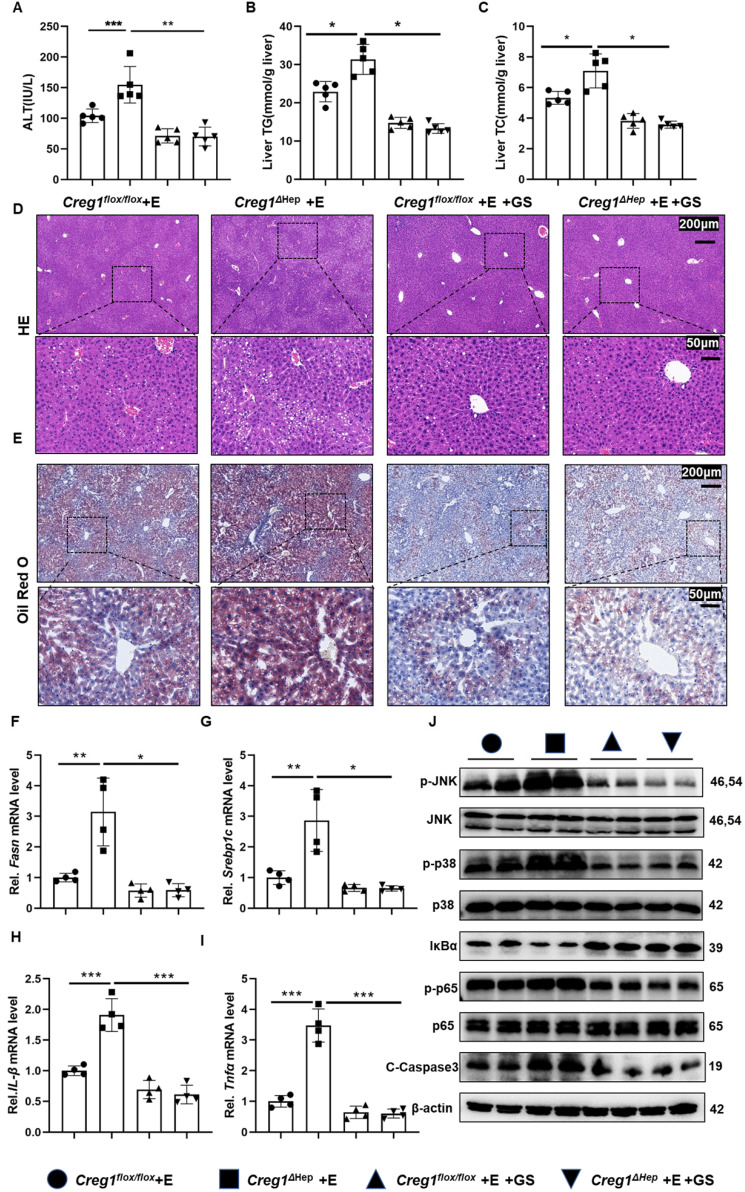
** Treatment with an ASK1 inhibitor abolishes alcoholic liver injury in *Creg1^∆hep^* mice. (A-J)** *Creg1^flox/flox^* and *Creg1^∆hep^* mice were injected with the ASK1 inhibitor GS4997 or vehicle (DMSO) before ethanol feeding (Gao-binge model). **(A)** The serum levels of ALT in the indicated groups (*n*=5 per group). **(B)** Hepatic triglyceride (TG) and **(C)** total cholesterol (TC) levels (*n*=5 per group). **(D)** Representative HE staining and **(E)** Oil Red O staining of liver tissues from the indicated groups. Scale bar = 200 µm; scale bar = 50 µm. **(F-I)** mRNA levels of *Fasn*, *Srebp1c, Il-1β* and *Tnfα* in the indicated groups (*n*=4 per group). **(G)** Protein levels of ASK1, JNK, p38, p65, IκBα, C-Casp3 and phosphorylated ASK1, JNK, p38, and p65 in liver tissues from the indicated groups. β-Actin served as the loading control. All data are represented as the mean ± SD. *p < 0.05, **p < 0.01 by two-tailed Student's t test.

To ascertain whether ASK1 mediates CREG1 function in ethanol-induced liver injury, a specific ASK1 inhibitor, GS-4997, was used. As shown in Figure 7A, GS-4997 reversed the exacerbation of ethanol-induced liver injury caused by CREG1 deficiency. We also observed that GS-4997 reduced the increases in hepatic TG and TC (Figure 7B, C). In addition, the accumulation of liver fat in ethanol-fed *Creg1^∆hep^* mice were reduced after GS-4997 treatment. (Figure 7D, E). GS-4997 abolished the exacerbation of liver fat accumulation and inflammatory responses induced by CREG1 deletion during ALD, as evidenced by decreased expression of genes encoding molecules related to lipid synthesis (*Fasn* and *Srebp1c*) and proinflammatory factors (*Il-1β* and *Tnfα*) (Figure 7F, G, H, I). Western blotting analysis showed that GS-4997 blocked activation of the NF-κB signalling pathway (Figure 7J). The protein levels C-caspase3 were downregulated by GS-4997. GS-4997 inhibited the hyperactivation of the JNK/p38 signalling pathway in ethanol-fed *Creg1^∆hep^* mice (Figure 7J). These observations suggest that deletion of CREG1 leads to an intensification of ethanol-induced liver injury that is inhibited by blocking the activity of ASK1.

## Discussion

**Figure 8:**
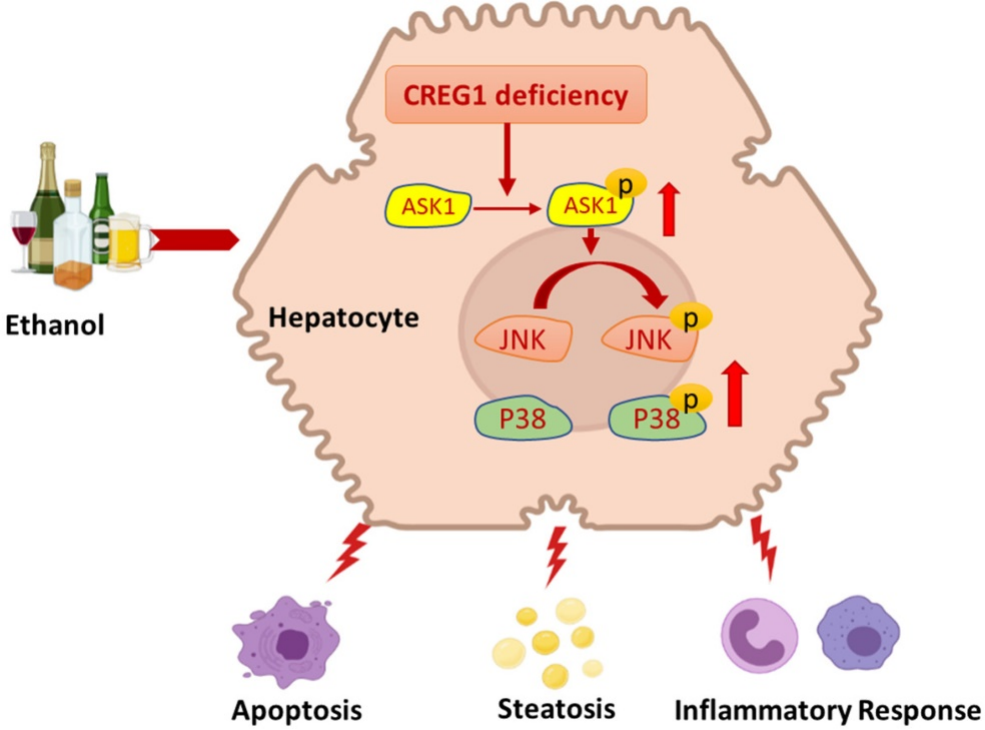
** Schematic representation of the role of the CREG1-ASK1-JNK/p38 axis in the pathogenesis of ALD.** Hepatocyte-specific knockout of CREG1 aggravated ethanol-induced liver injury, apoptosis, steatosis and inflammation through the ASK1-JNK/p38 signalling pathway.

Alcohol misuse is a significant cause of liver-related death, and abstinence from alcohol is the mainstay of treatment for this disease. In addition to lifestyle changes for patients with heavy alcohol dependence, pharmacological/genetic interventions are the best way to combat the difficulties encountered when suggesting lifestyle changes for patients with alcohol addiction. In the present study, with a tissue-specific loss-of-function approach, hepatic CREG1 was shown to be a protective factor in ALD. Hepatocyte-specific CREG1 deficiency aggravated ethanol-induced liver injury, apoptosis, steatosis and inflammation. Further research demonstrated that CREG1 inhibits p38/JNK activation by suppressing ASK1. These findings suggest that the CREG1-ASK1-JNK/P38 axis is positively involved in the regulation of ALD and could be a possible therapeutic target (Figure 8).

The increased protein levels of CREG1 during ALD indicate its possible function in ALD pathology. Alcohol intake can cause epigenetic alterations in hepatocytes, such as changes in acetylation, hypomethylation of DNA, and alterations of miRNAs. These modifications can be induced by alcohol-induced oxidative stress, which results in altered recruitment of transcriptional machinery and abnormal gene expression. It was reported that the abundance and activity of CREG1 are affected by epigenetic modifications and posttranslational modifications, including DNA methylation, noncoding RNA-related mechanisms and glycosylation 28-30. Therefore, we hypothesized that alcohol causes epigenetic modifications of CREG1 that increase CREG1 protein expression. We speculated that the elevated levels of CREG1 protein in ALD mice and ALD patients are a consequence of altered upstream signalling pathways. Given that overexpression of CREG1 inhibits activation of ASK1 and may protect the liver from alcohol-induced liver injury, the moderate increase in CREG1 may be a compensatory mechanism to protect hepatocytes from alcohol-induced damage.

ALD is a complex process that results in a wide spectrum of hepatic disorders, from steatosis to cirrhosis, and eventually leads to hepatocellular carcinoma. Apoptosis, steatosis, and inflammation are key drivers of ethanol-induced liver injury 1. In a previous study, CREG attenuated atherosclerotic endothelial apoptosis via the VEGF/PI3K/AKT pathway and protected retinal cells and human smooth muscle cells against apoptosis by inhibiting P38/MAPK and JNK/MAPK signalling 31. ASK1 is a ubiquitously expressed member of the MAPKK family and a key regulator of apoptosis, necrosis and inflammation. We found that CREG1 regulates apoptosis by targeting ASK1-JNK/p38 signalling in ALD.

Among the outcomes of chronic alcohol consumption, steatosis has been considered a reversible pathological change in the liver. As a result of chronic hepatic steatosis, the liver is more vulnerable to advanced ALD-related conditions, such as steatohepatitis, hepatic fibrosis and cirrhosis. A previous study reported that CREG1 inhibited HFD-induced hepatic steatosis and metabolic disorders 18. Moreover, CREG1 also stimulates brown adipocyte formation and ameliorates diet-induced obesity in mice 32. In the present study, our data demonstrated that *Creg1^∆hep^* mice develop more severe steatosis in response to ethanol feeding. The lipid metabolism-related AMPK and mTOR signalling pathways were analysed, and phosphorylation of AMPK was increased, whereas that of mTOR was decreased. SREBP1, PPARα, and PPARγ are key factors regulating TG production and consumption in response to ethanol 33, 34. These findings show that for both non-alcoholic and alcoholic fatty liver disease, CREG1 might be an attractive therapeutic target.

In addition to steatosis, inflammation also plays a significant role in the development of ALD. Several proinflammatory signals, including chemokines, cytokines, and lipid messengers, contribute to exacerbating alcohol-induced liver damage. Several studies have shown that CREG1 protects against cell death and inflammation during hepatic I/R injury 17 and myocardial infarction 35. In our study, hepatic CREG1 deficiency exacerbated the ethanol-induced inflammatory response by promoting NF-κB activation. This result indicates that CREG1 can protect against ethanol-induced liver injury by regulating inflammation.

The MAPK signalling pathway has profound biological functions, participating in processes including inflammation, cell cycle regulation, cell death and lipid metabolism. In a MAPK cascade, an activating MAPK activates a second MAPK, which in turn activates a terminal MAPK (ERK, p38, JNK) to amplify and modulate the signals 36. A previous study demonstrated that CREG blocked MAPK activation to protect against cell death and inflammation during hepatic I/R injury 17. In addition, CREG1 inhibited HFD-induced hepatic steatosis and metabolic disorders by interacting with MAPK/ASK1-JNK1 18. The current study showed that CREG1 improved ethanol-induced liver injury, apoptosis, steatosis, and inflammation by targeting ASK1-JNK/p38 signalling.

ASK1 and TAK1 are upstream regulators of p38 and JNK in response to various stressors. In our study, CREG1 deficiency increased the phosphorylation of ASK1 but not that of TAK1. Previous studies have shown that multiple downstream signalling pathways, such as the NF-κB, MAPK/JNK, and MAPK/p38 pathways, are regulated by ASK1, causing liver inflammation and apoptosis in various diseases, including ALD, non-alcoholic fatty liver disease, hepatic I/R injury, and cancer 37, 38. Additionally, we investigated that overactivation of JNK and p38 after CREG1 deletion is dependent on upstream ASK1 signalling. Interestingly, overexpression of CREG1 alone was not sufficient to regulate the ASK1 signalling in the absence of alcohol *in vitro* experiments. It seems that the regulation of the ASK1 signalling pathway by CREG1 needs to be mediated by other alcohol-regulatory proteins, which deserves further exploration in future experiments. Importantly, ASK1 inhibition prevented apoptosis, steatosis, and inflammation in ethanol-fed *Creg1^∆hep^* mouse livers. Based on all these findings, ASK1 serves as a downstream target of CREG1 and mediates the protective function of CREG1 in ALD. Here, our findings suggest that increasing CREG1 abundance in hepatocytes could be an effective treatment for ALD.

Aging as a risk factor has been shown to modify the development and progression of ALD 39. In our previous study, aging increases the susceptibility of alcohol-induced liver injury by downregulating sirtuin 1 (SIRT1) in hepatocytes and hepatic stellate cells, and the SIRT1-C/EBPα-miR-223 axis in neutrophils 7, 8. The ASK1-signalosome network is a major center of stress signals that plays multiple roles in promotion of senescence, aging and diseases of oxidative stress 40. It is reported that p38MAPK mediates the stress signal to induce senescence 41. Previous studies have reported that several genes that regulate aging are involved in the development of ALD, such as SIRT1, SIRT2 and SIRT6 42-44. Importantly, CREG1 is also involved in the ageing process 10, 11. Therefore, the CREG1-ASK1-JNK/p38 axis likely plays an important role in ageing and alcoholic liver injury, which deserves further studies. In summary, the present study demonstrated that CREG1 plays an important role in ALD and exerts its function in a matter dependent on the ASK1-JNK/p38 pathway. These findings highlight for the first time that targeting CREG1 might be a promising therapeutic strategy for ALD prevention and treatment.

## Supplementary Material

Supplementary figures and tables.

## Abbreviations

ALD: Alcohol-associated liver disease
ALT: alanine aminotransferase
AST: aspartate aminotransferase
ASK1: apoptosis signal-regulating kinase 1
CREG1: cellular repressor of E1A-stimulated genes 1
*Creg1^∆hep^*: hepatocyte-specific CREG1 knockout
*Creg1^floxl/flox^* mice: *Creg1^∆hep^* littermates
HE: haematoxylin and eosin
JNK: c-Jun N-terminal kinase
MAPK: mitogen-activated protein kinase
qRT-PCR: quantitative real-time polymerase chain reaction
TG: triglyceride
TC: total cholesterol
TUNEL: terminal deoxynucleotidyl transferase dUTP nick end labelling

## References

[1] Seitz HK, Bataller R, Cortez-Pinto H, Gao B, Gual A, Lackner C (2018). Alcoholic liver disease. Nat Rev Dis Primers.

[2] Gao B, Bataller R (2011). Alcoholic liver disease: pathogenesis and new therapeutic targets. Gastroenterology.

[3] Louvet A, Mathurin P (2015). Alcoholic liver disease: mechanisms of injury and targeted treatment. Nat Rev Gastroenterol Hepatol.

[4] Ghobrial G, Araujo L, Jinwala F, Li S, Lee LY (2018). The Structure and Biological Function of CREG. Front Cell Dev Biol.

[5] Sacher M, Di Bacco A, Lunin VV, Ye Z, Wagner J, Gill G (2005). The crystal structure of CREG, a secreted glycoprotein involved in cellular growth and differentiation. Proc Natl Acad Sci U S A.

[6] Kim IH, Kisseleva T, Brenner DA (2015). Aging and liver disease. Curr Opin Gastroenterol.

[7] Ramirez T, Li YM, Yin S, Xu MJ, Feng D, Zhou Z (2017). Aging aggravates alcoholic liver injury and fibrosis in mice by downregulating sirtuin 1 expression. J Hepatol.

[8] Ren R, He Y, Ding D, Cui A, Bao H, Ma J (2021). Aging exaggerates acute-on-chronic alcohol-induced liver injury in mice and humans by inhibiting neutrophilic sirtuin 1-C/EBPalpha-miRNA-223 axis. Hepatology.

[9] Ma J, Cao H, Rodrigues RM, Xu M, Ren T, He Y (2020). Chronic-plus-binge alcohol intake induces production of proinflammatory mtDNA-enriched extracellular vesicles and steatohepatitis via ASK1/p38MAPKalpha-dependent mechanisms. JCI Insight.

[10] Moolmuang B, Tainsky MA (2011). CREG1 enhances p16(INK4a) -induced cellular senescence. Cell Cycle.

[11] Hashimoto M, Goto A, Endo Y, Sugimoto M, Ueda J, Yamashita H (2021). Effects of CREG1 on Age-Associated Metabolic Phenotypes and Renal Senescence in Mice. Int J Mol Sci.

[12] Liu J, Qi Y, Chao J, Sathuvalli P, L YL, Li S (2021). CREG1 promotes lysosomal biogenesis and function. Autophagy.

[13] Song H, Tian X, Liu D, Liu M, Liu Y, Liu J (2021). CREG1 improves the capacity of the skeletal muscle response to exercise endurance via modulation of mitophagy. Autophagy.

[14] Han Y, Wu G, Deng J, Tao J, Guo L, Tian X (2010). Cellular repressor of E1A-stimulated genes inhibits human vascular smooth muscle cell apoptosis via blocking P38/JNK MAP kinase activation. J Mol Cell Cardiol.

[15] Song H, Yan C, Tian X, Zhu N, Li Y, Liu D (2017). CREG protects from myocardial ischemia/reperfusion injury by regulating myocardial autophagy and apoptosis. Biochim Biophys Acta Mol Basis Dis.

[16] Sun M, Tian X, Liu Y, Zhu N, Li Y, Yang G (2015). Cellular repressor of E1A-stimulated genes inhibits inflammation to decrease atherosclerosis in ApoE(-/-) mice. J Mol Cell Cardiol.

[17] Yang L, Wang W, Wang X, Zhao J, Xiao L, Gui W (2019). Creg in Hepatocytes Ameliorates Liver Ischemia/Reperfusion Injury in a TAK1-Dependent Manner in Mice. Hepatology.

[18] Zhang QY, Zhao LP, Tian XX, Yan CH, Li Y, Liu YX (2017). The novel intracellular protein CREG inhibits hepatic steatosis, obesity, and insulin resistance. Hepatology.

[19] Song Q, Chen Y, Wang J, Hao L, Huang C, Griffiths A (2020). ER stress-induced upregulation of NNMT contributes to alcohol-related fatty liver development. J Hepatol.

[20] Wang Z, Dou X, Li S, Zhang X, Sun X, Zhou Z (2014). Nuclear factor (erythroid-derived 2)-like 2 activation-induced hepatic very-low-density lipoprotein receptor overexpression in response to oxidative stress contributes to alcoholic liver disease in mice. Hepatology.

[21] Bertola A, Mathews S, Ki SH, Wang H, Gao B (2013). Mouse model of chronic and binge ethanol feeding (the NIAAA model). Nat Protoc.

[22] Sanz-Garcia C, Poulsen KL, Bellos D, Wang H, McMullen MR, Li X (2019). The non-transcriptional activity of IRF3 modulates hepatic immune cell populations in acute-on-chronic ethanol administration in mice. J Hepatol.

[23] Wang S, Ni HM, Chao X, Ma X, Kolodecik T, De Lisle R (2020). Critical Role of TFEB-Mediated Lysosomal Biogenesis in Alcohol-Induced Pancreatitis in Mice and Humans. Cell Mol Gastroenterol Hepatol.

[24] Ambade A, Lowe P, Kodys K, Catalano D, Gyongyosi B, Cho Y (2019). Pharmacological Inhibition of CCR2/5 Signaling Prevents and Reverses Alcohol-Induced Liver Damage, Steatosis, and Inflammation in Mice. Hepatology.

[25] Inoki K, Kim J, Guan KL (2012). AMPK and mTOR in cellular energy homeostasis and drug targets. Annu Rev Pharmacol Toxicol.

[26] Bian Z, Cai J, Shen DF, Chen L, Yan L, Tang Q (2009). Cellular repressor of E1A-stimulated genes attenuates cardiac hypertrophy and fibrosis. J Cell Mol Med.

[27] Xu L, Liu JM, Chen LY (2004). CREG, a new regulator of ERK1/2 in cardiac hypertrophy. J Hypertens.

[28] Di Bacco A, Gill G (2003). The secreted glycoprotein CREG inhibits cell growth dependent on the mannose-6-phosphate/insulin-like growth factor II receptor. Oncogene.

[29] Liu Y, Tian X, Liu S, Liu D, Li Y, Liu M (2020). DNA hypermethylation: A novel mechanism of CREG gene suppression and atherosclerogenic endothelial dysfunction. Redox Biol.

[30] Wang J, Yan CH, Li Y, Xu K, Tian XX, Peng CF (2013). MicroRNA-31 controls phenotypic modulation of human vascular smooth muscle cells by regulating its target gene cellular repressor of E1A-stimulated genes. Exp Cell Res.

[31] Zhang TZ, Hua T, Han LK, Zhang Y, Li GY, Zhang QL (2018). Antiapoptotic role of the cellular repressor of E1A-stimulated genes (CREG) in retinal photoreceptor cells in a rat model of light-induced retinal injury. Biomed Pharmacother.

[32] Hashimoto M, Kusudo T, Takeuchi T, Kataoka N, Mukai T, Yamashita H (2019). CREG1 stimulates brown adipocyte formation and ameliorates diet-induced obesity in mice. FASEB J.

[33] Ceni E, Mello T, Galli A (2014). Pathogenesis of alcoholic liver disease: role of oxidative metabolism. World J Gastroenterol.

[34] Ji C, Chan C, Kaplowitz N (2006). Predominant role of sterol response element binding proteins (SREBP) lipogenic pathways in hepatic steatosis in the murine intragastric ethanol feeding model. J Hepatol.

[35] Peng CF, Han YL, Jie D, Yan CH, Jian K, Bo L (2011). Overexpression of cellular repressor of E1A-stimulated genes inhibits TNF-alpha-induced apoptosis via NF-kappaB in mesenchymal stem cells. Biochem Biophys Res Commun.

[36] Fang JY, Richardson BC (2005). The MAPK signalling pathways and colorectal cancer. Lancet Oncol.

[37] Ogier JM, Nayagam BA, Lockhart PJ (2020). ASK1 inhibition: a therapeutic strategy with multi-system benefits. J Mol Med (Berl).

[38] Cargnello M, Roux PP (2011). Activation and function of the MAPKs and their substrates, the MAPK-activated protein kinases. Microbiol Mol Biol Rev.

[39] Crabb DW, Im GY, Szabo G, Mellinger JL, Lucey MR (2020). Diagnosis and Treatment of Alcohol-Associated Liver Diseases: 2019 Practice Guidance From the American Association for the Study of Liver Diseases. Hepatology.

[40] Papaconstantinou J (2019). The Role of Signaling Pathways of Inflammation and Oxidative Stress in Development of Senescence and Aging Phenotypes in Cardiovascular Disease. Cells.

[41] Freund A, Orjalo AV, Desprez PY, Campisi J (2010). Inflammatory networks during cellular senescence: causes and consequences. Trends Mol Med.

[42] Kim HG, Huang M, Xin Y, Zhang Y, Zhang X, Wang G (2019). The epigenetic regulator SIRT6 protects the liver from alcohol-induced tissue injury by reducing oxidative stress in mice. J Hepatol.

[43] Ren R, Wang Z, Wu M, Wang H (2020). Emerging Roles of SIRT1 in Alcoholic Liver Disease. Int J Biol Sci.

[44] Zhang Y, Long X, Ruan X, Wei Q, Zhang L, Wo L (2021). SIRT2-mediated deacetylation and deubiquitination of C/EBPbeta prevents ethanol-induced liver injury. Cell Discov.

