# Supplementary material for: Hepatocyte-specific deletion of cellular repressor of E1A-stimulated genes 1 exacerbates alcohol-induced liver injury by activating stress kinases
Source: Int J Biol Sci. 2022 Jan 31;18(4):1612–26. doi: 10.7150/ijbs.67852 (PMC8898367; doi:10.7150/ijbs.67852)
Supplement: Supplementary file 1 — Supplementary figures and tables. [file ijbsv18p1612s1.pdf]

Supplementary Figure 1

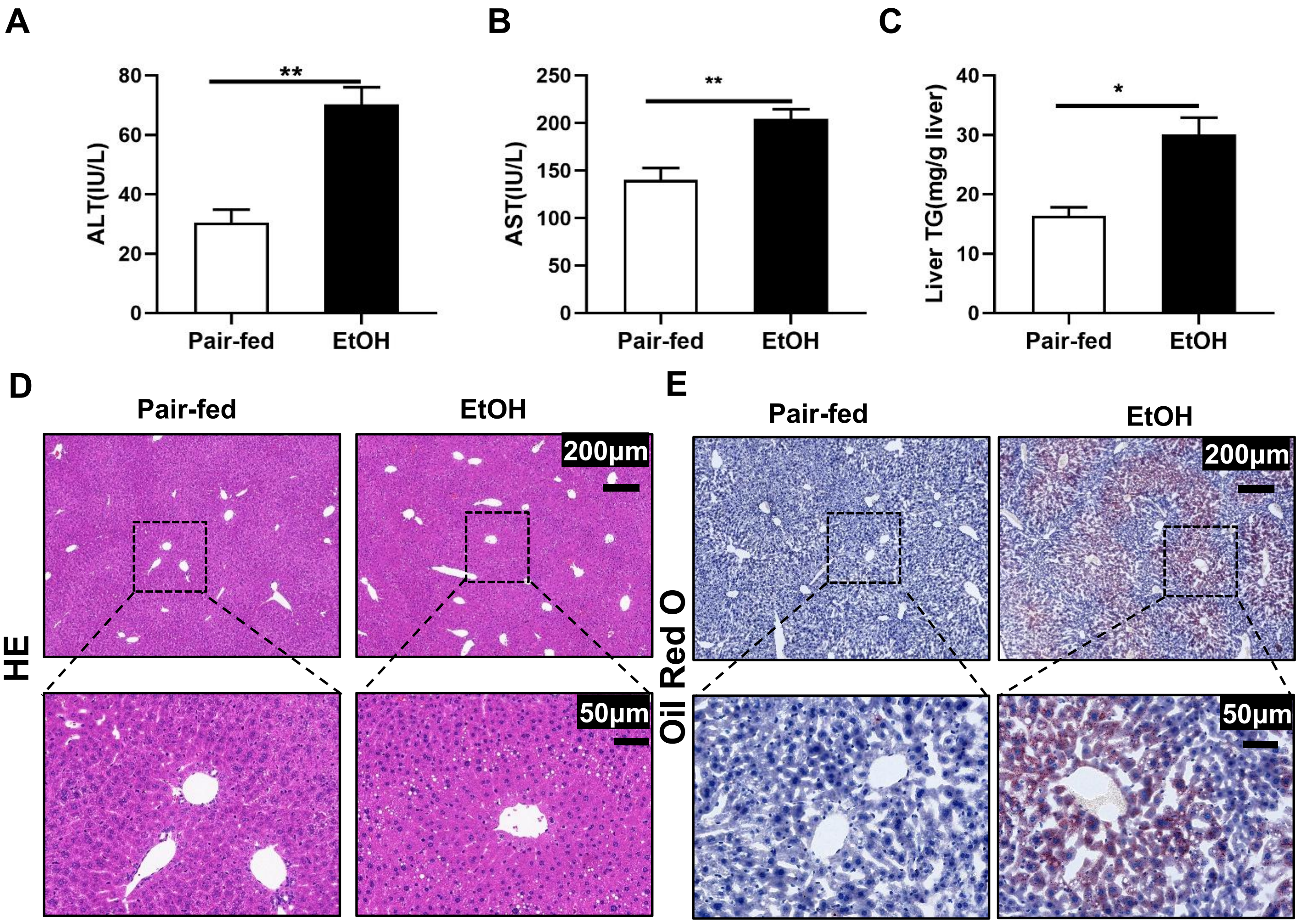

Supporting Figure 1. Establishment of Gao-binge model in mice.(A) Serum ALT and (B) AST level of wt mice under NIAAA model (n=4-6 Per group). (C) Hepatic triglyceride (TG) levels. Representative images of (D) HE and (E) Oil red O staining of liver tissues in pair-fed and ethanol-fed mice. All data are represented as the mean ± SD. *p < 0.05, **p < 0.01.

Supplementary Figure 2

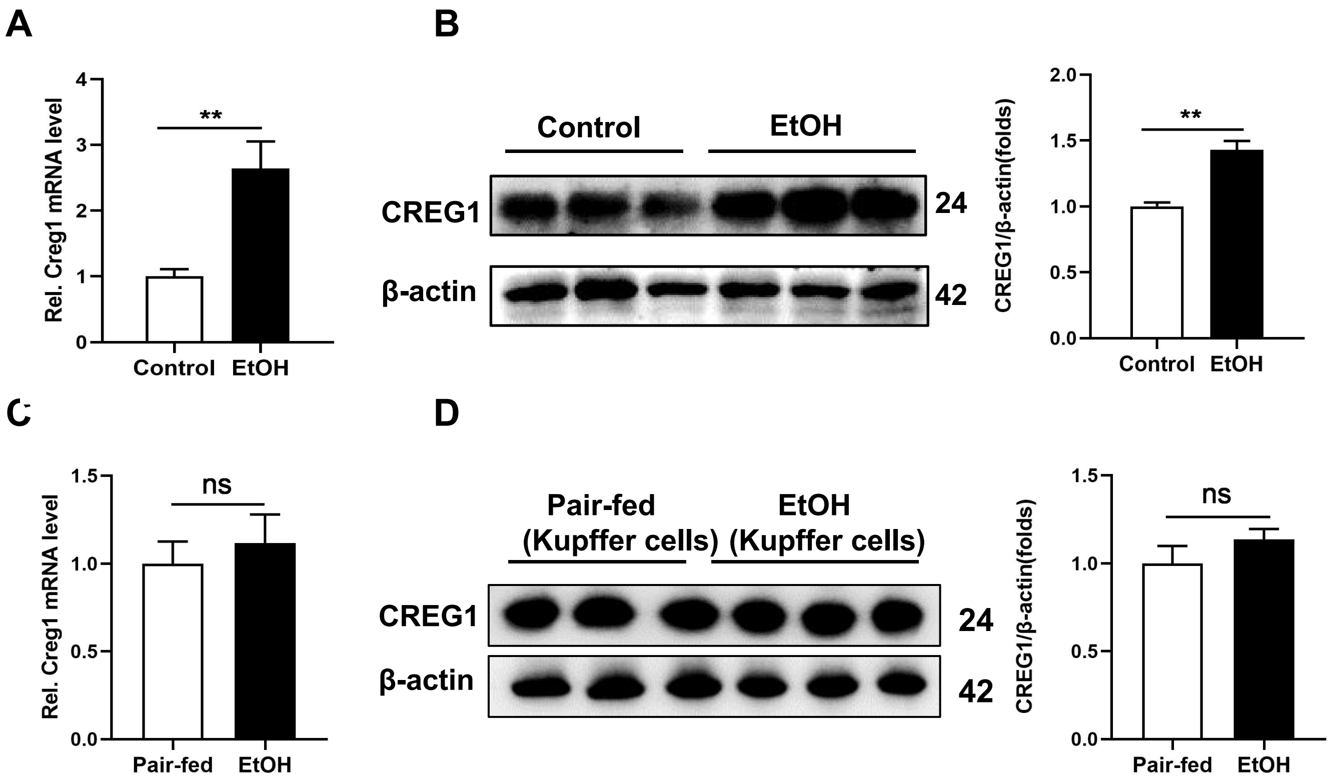

Supporting Figure 2. CREG1 increased in AML12 cells but not in Kupffer cells of mice after ethanol treatment.(A)mRNA levels and (B) protein levels of CREG1 in AML12 cells with ethanol treatment, (C)mRNA levels and (D) protein levels of CREG1 in Kupffer cells of mice after alcohol consumption,β-actin served as the loading control. All data are represented as the mean ± SD. *p < 0.05, **p < 0.01. All data are represented as the mean ± SD. *p < 0.05, **p < 0.01.

Supplementary Figure 3

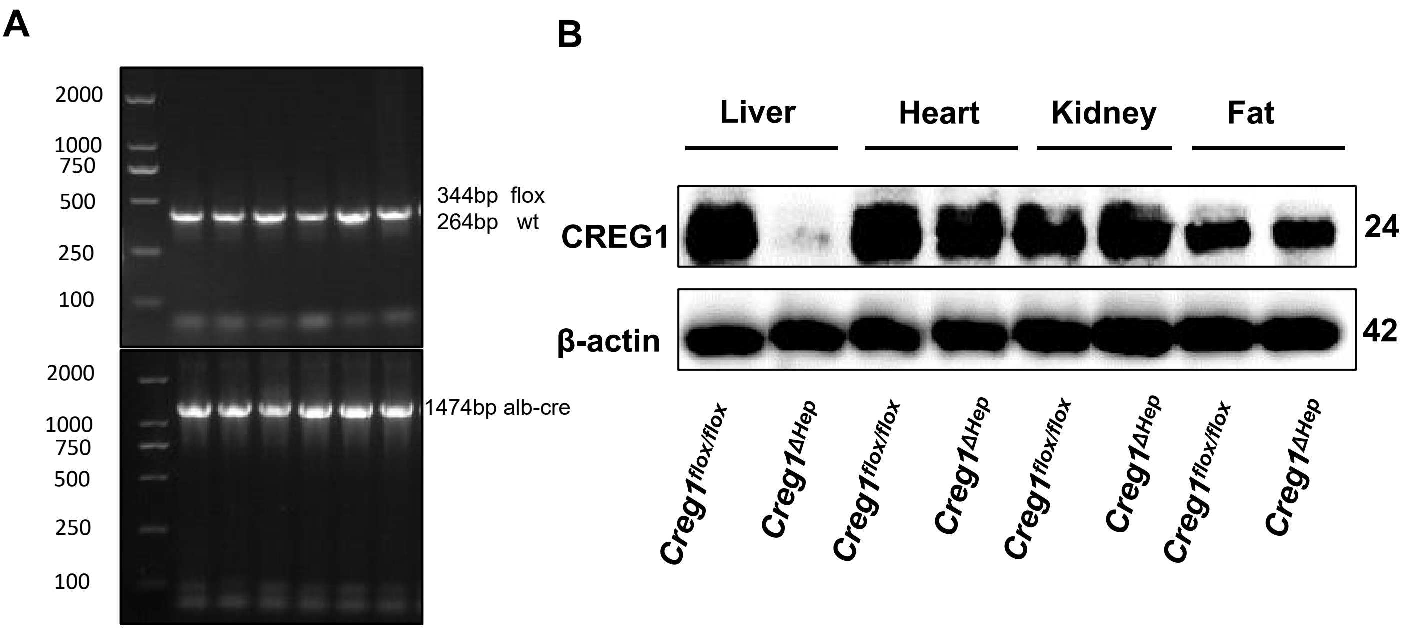

Supporting Figure 3. The generation of hepatocyte specific-knockout CREG1 mouse strain. (A) Phenotype identification of control mice(*Creg1*$^{flox/flox}$) and hepatocyte specific-knockout CREG1 mice(*Creg1*$^{\Delta hep}$).(B)Protein levels of CREG1 in major organ from *Creg1*$^{flox/flox}$ and *Creg1*$^{\Delta hep}$ mice. β-actin served as the loading control.

Supplementary Figure 4

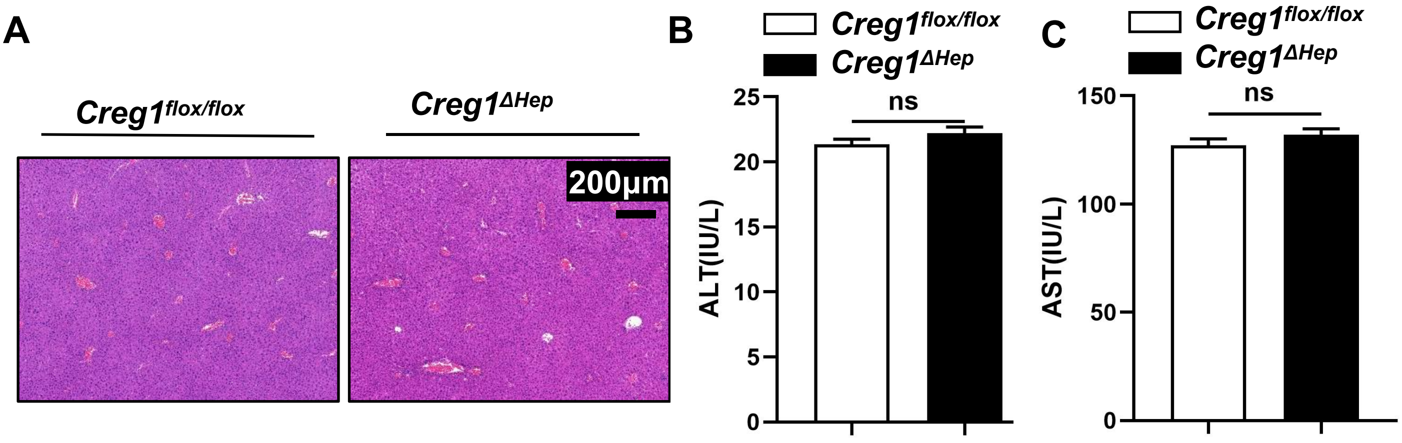

Supplementary Figure 4.. Hepatocyte-specific Knockout CREG1 deficiency does not cause liver injury in normal diet.
(A)Representative HE staining of liver tissues from *Creg1*$^{flox/flox}$ and *Creg1*$^{\Delta hep}$ mice in normal diet. The serum levels of (B) ALT and (C) AST in *Creg1*$^{flox/flox}$ and *Creg1*$^{\Delta hep}$ mice in normal diet (n = 6 Per group). All data are represented as the mean ± SD. $^*p < 0.05$, $^{**}p < 0.01$.

Supplementary Figure 5

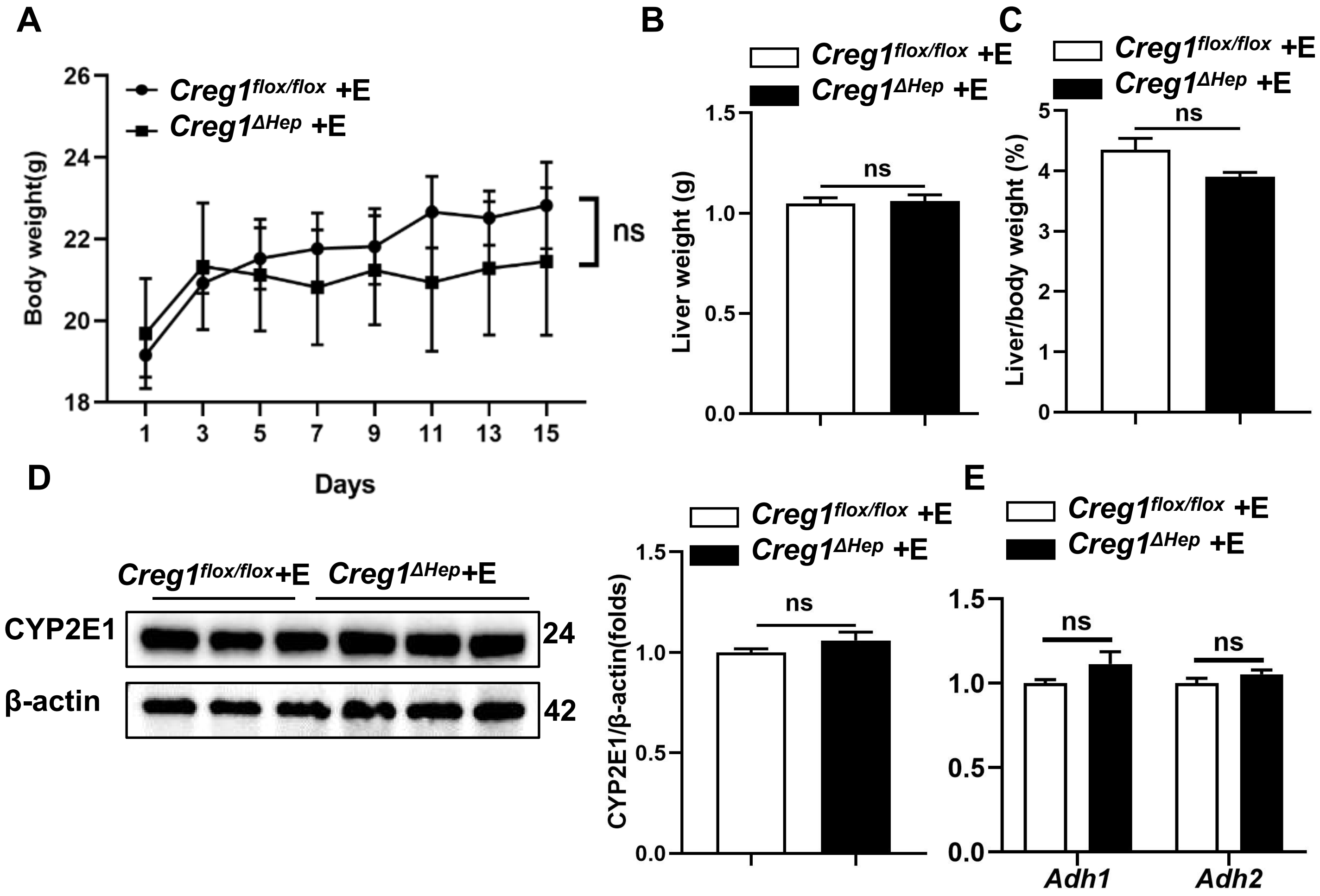

Supplementary Figure 5. Body weight and ethanol consumption in ethanol-fed *Creg1*$^{flox/flox}$ and *Creg1*$^{\Delta hep}$ mice. (A-E) *Creg1*$^{flox/flox}$ and *Creg1*$^{\Delta hep}$ mice were ethanol-fed for 10 days plus a single binge of ethanol (NIAAA model). (A) Body weights, (B) liver weights and (C) liver to body weight ratios of *Creg1*$^{flox/flox}$ and *Creg1*$^{\Delta hep}$ mice under the NIAAA model (n = 6 Per group). (D) The protein expression of CYP2E1 (*n*=3 per group) (E) Levels of ethanol metabolism-related gene expression t (*n*=3 per group). β-actin served as the loading control. All data are represented as the mean ± SD. *p < 0.05, **p < 0.01. CYP2E1, cytochrome P450 2E1, *Adh1 ,* alcohol dehydrogenase 1, A*dh*2 ,alcohol dehydrogenase 2.

Supplementary Figure 6

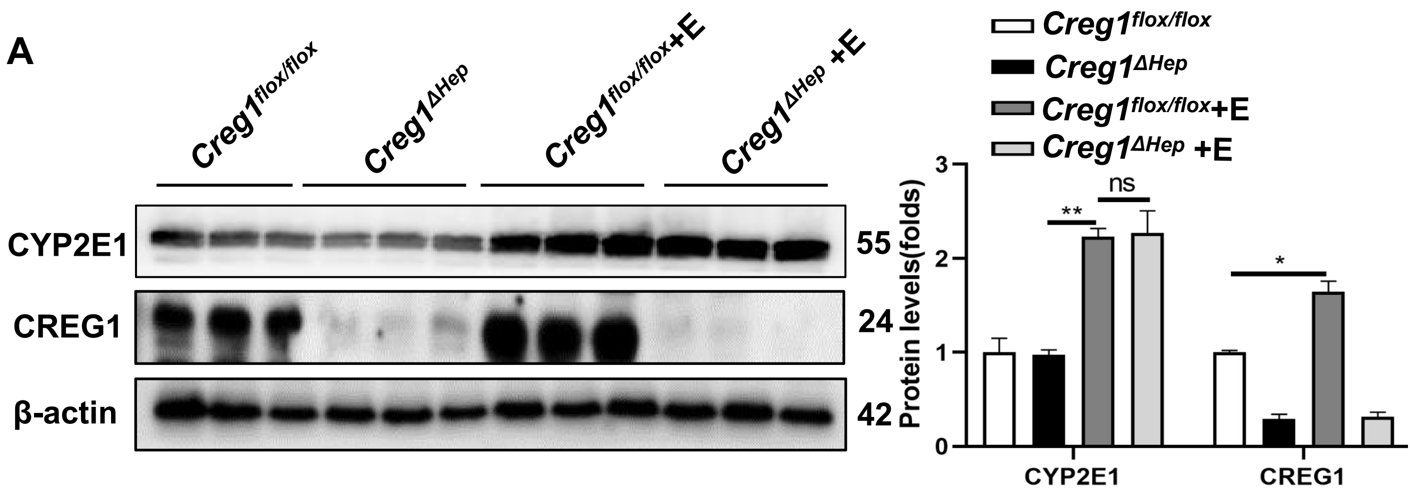

Supplementary Figure 6. CYP2E1 and CREG1 expression in *Creg1*$^{flox/flox}$ and *Creg1*$^{\Delta hep}$ mice under traditional model. (A)The protein expression of CYP2E1 and CREG1.(*n*=3 per group). β-actin served as the loading control. All data are represented as the mean ± SD. *p < 0.05, **p < 0.01.

Supplementary Figure 7

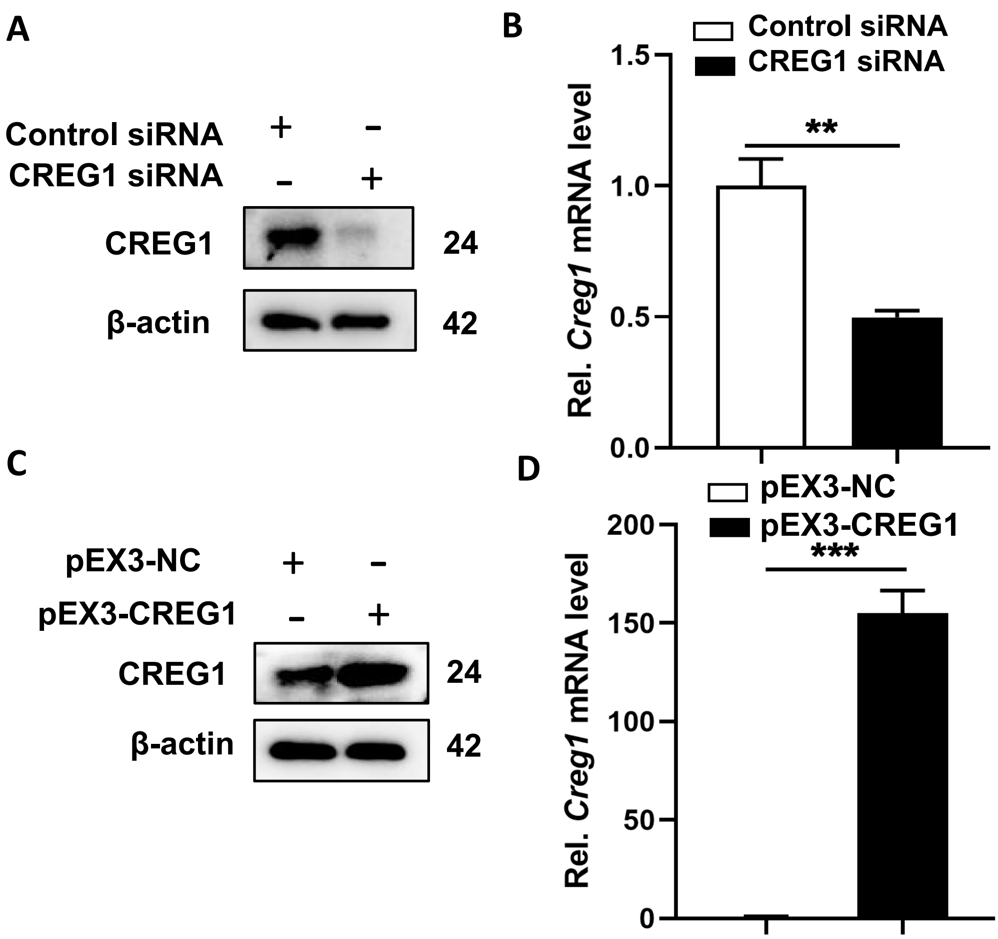

Supplementary Figure 7 . Silence or overexpression of CREG1 in AML12 cells. (A) The protein levels and (B) mRNA levels of CREG1 in AML12 cells transfected with control siRNA or CREG1 siRNA. (C)) The protein levels and (D) mRNA levels of CREG1 in AML12 cells transfected with pEX3-NC or pEX-3-CREG1. β-actin served as the loading control. All data are represented as the mean ± SD. *$p < 0.05$, **$p < 0.01$.

Supplementary Table S1. The healthy individual and intoxicated patients information.

Healthy			intoxicated patients			
Age	ALT	AST	Age	ALT	AST	Ethanol(ml)
55	11	17	35	37	26	250
58	24	18	45	48	54	200
46	12	20	35	74	62	300
65	11	17	23	39	26	200
32	17	15	70	45	30	200
57	14	15	40	75	44	500
65	23	20	23	34	22	300
85	20	18	21	28	23	300
57	17	18	26	24	27	250
32	12	16	47	36	42	300
32	13	15	22	23	46	200
71	14	17	37	25	25	350
41	15	26	35	77	54	300
62	20	21	48	21	20	150
58	16	31	40	27	26	200
55	18	17	52	154	115	250
41	10	13	25	39	28	500
50	13	16	21	49	44	350
79	21	26	37	24	22	250
28	17	22	46	42	33	150
64	11	20	19	21	22	350
57	33	21	34	36	20	200
53	18	18	29	37	25	500
47	24	23	30	33	21	250
57	23	23	21	21	20	200
24	17	15	38	44	17	250
56	29	19	19	18	28	250
29	16	18	34	59	29	300
35	15	15	36	66	94	250
48	31	19	43	37	27	250
37	16	21	30	38	30	500
40	11	14	30	45	32	500
69	20	18	22	24	22	300
75	14	18	30	86	92	200
60	13	19	32	60	33	200
66	15	19	22	34	56	400
62	18	19	35	31	22	250
31	18	34	26	21	22	250
65	18	17	27	40	29	250
28	24	30	50	25	27	500
79	20	21	66	32	23	150
50	16	42	30	32	30	300
26	16	14	31	35	28	300

Supplementary Table S2.The antibodies information.

Antibody	Cat NO.	Manufacturer
CREG1	ab233282/sc-100695	Abcam/Santa cruz
BAX	ab32503	Abcam
BCL-2	ab182858	Abcam
C-Casp3	ab214430	Abcam
p-mTOR	5536S	CST
mTOR	66888-1-Ig	Proteintech
p-AMPK	2535S	CST
AMPK	66536-1-Ig	Proteintech
SREBP1	66875-1-Ig	Proteintech
PPARα	15540-1-AP	Proteintech
PPARγ	2435S	CST
p65	9936T	CST
p-p65	9936T	CST
IKKβ	9936T	CST
p-IKKβ	9936T	CST
IκBα	9936T	CST
ASK1	AF6477	Affinity
p-ASK1	AF3477	Affinity
TAK1	AF7616	Affinity
p-TAK1	AF4379	Affinity
JNK	9252T	CST
p-JNK	4668S	CST
p38	8690S	CST
p-p38	9216S	CST
ERK	4695S	CST
p-ERK	4370S	CST
CYP2E1	19937-1-AP	Proteintech
F4/80	70076S	CST
MPO	ab208670	Abcam
β-actin	TA-09	ZSGB

Supplementary Table S3. The primer information.

Gene	Forward primer	Reverse primer
β-actin	CACCCGCGATACAACCTTC	CCCATACCCACCATCACACC
Creg1	GTGGCACTACTGGTGTCGC	CGCGCACCTCCTTTATTGTG
Srebp1c	AACGTCACTTCCAGCTAGAC	CCACTAAGGTGCCTACAGAGC
Fasn	TTGGCCCAGAACTCCTGTAG	CTCGCTTGTCGTCTGCCT
Srebp2	CCCTATTCCATTGACTCTGAGC	GAGTCCGGTTCATCCTTGAC
Hmgcr	CACAATAACTTCCCAGGGGT	GGCCTCCATTTAGATCCG
Chrebp	ACAAAAAGCGGCTCCGTAAGTCC	GGGGGCGGTAATTGGTGAAGAAA
Pparα	AACATCGAGTGTCGAATATGTGG	CCGAATAGTTCGCCGAAAGAA
Tnfα	CAGGCGGTGCCTATGTCTC	CGATCACCCCGAAGTTCAGTAG
IL-6	CTGCAAGAGACTTCCATCCAG	AGTGGTATAGACAGGTCTGTTGG
IL-1β	GAAATGCCACCTTTTGACAGTG	TGGATGCTCTCATCAGGACAG
F4/80	CTGCACCTGTAAACGAGGCTT	GCAGACTGAGTTAGGACCACAA
Ly6g	TGCGTTGCTCTGGAGATAGA	CAGAGTAGTGGGGCAGATGG
Ccl2	TAAAAACCTGGATCGGAACCAAA	GCATTAGCTTCAGATTTACGGGT
Adh1	CCATCGAGGACATAGAAGTCGC	TGGTTTCACACAAGTCACCCC
Adh2	TGGCAGTCCCCTTTGCATT	ACTACCGGGAAGAGAGCTTTC
